# Seroprevalence of *Brucella abortus* in cattle in Southern Lebanon using different diagnostic tests

**DOI:** 10.14202/vetworld.2020.2234-2242

**Published:** 2020-10-28

**Authors:** Hussein Hassan, Ali Salami, Ghassan Ghssein, Jeanne El-Hage, Nada Nehme, Rana Awada

**Affiliations:** 1 Department of Natural Sciences, School of Arts and Sciences, Lebanese American University, Lebanon; 2 Rammal Hassan Rammal Research Laboratory, PhyToxE Research Group, Faculty of Sciences (V), Lebanese University, Nabatieh, Lebanon; 3 Animal Health Laboratory, Lebanese Agricultural Research Institute, Fanar, Lebanon; 4 Department of Veterinary Sciences, Faculty of Agronomy, Lebanese University, Dekwaneh, Lebanon

**Keywords:** Brucellosis, *Brucella abortus*, diagnostic tests, prevalence, Southern Lebanon

## Abstract

**Background and Aim:**

Brucellosis is endemic zoonotic and highly contagious bacterial disease. Recently, several brucellosis cases were reported in Lebanon, causing significant economic losses; however, no study was done so far on farms located in the southern part of the country. Thus, the aim of our study was to estimate the prevalence of *Brucella abortus* in South Lebanon using three different serological tests in the diagnosis of brucellosis in cattle.

**Materials and Methods:**

Seventeen farms from 14 locations in Southern Lebanon were selected. Two hundred and three bovine blood samples of different ages, and 121 milk samples collected from older than 2 years cattle were tested using different serological tests: Rose Bengal test (RBT), milk ring test (MRT), indirect enzyme-linked immunosorbent assay (I-ELISA), and confirmed with competitive ELISA (C-ELISA).

**Results:**

Results revealed that approximately 15.3% (confidence interval [CI] 95 10.3-20.2%) and 15.7% (CI 95 9.2-22.2%) of samples were positive using RBT and MRT, respectively. This percentage was significantly higher when using I-ELISA (18.3%) (CI 95 12.9-23.5%) and C-ELISA (18.7%) (CI 95 9.8-27.5%). Among used diagnostic tests, our results showed that ELISA was more accurate for the detection of brucellosis, especially since it detects the late stages of the infection, which is characterized by the presence of immunoglobulin G. The seroprevalence of brucellosis was higher among females. All positive tests were of cattle Holstein breed older than 2 years. Tyre and Jezzine cities had a higher significance in bovine brucellosis than Saida. A positive correlation between human and cattle brucellosis was found.

**Conclusion:**

Our results showed that bovine brucellosis is prevalent in southern Lebanon. Lack of research, in addition to little feedback of occurring illness or symptoms, creates a gap in helping to control the spread of the disease.

## Introduction

Middle East is still considered an endemic region for Brucellosis according to the World Organization for Animal Health [1]. It is considered as a Category B with low mortality and moderate morbidity levels in Bioterrorism agents [2]. One study reported that frequency of the human incidences increased during spring through summertime [3], while another study indicated that the increase of infection occurs in summertime and autumn due to the breeding season and ambient temperature [4].

Brucellosis is a zoonotic disease where the infection causes an illness among humans [5]. This disease is caused by *Brucella* spp. from Brucellaceae family within *Alphaproteobacteria* class that includes different species and several biovars; thus, affecting a wide range of animals [6]. Cattle show an important affinity to *Brucella abortus* species that can infect sheep, goats, swine, elks, bison, camels, and humans.

Brucellosis is a systemic infection that can affect any organ, thus, causing neurological, cardiac, respiratory, and gastrointestinal problems [7,8]. In humans, bacteremia occurs for a longer period than in animals, and the reticuloendothelial system, musculoskeletal tissues, and gastro-urinary system are the targets [9-11]. Farmers, abattoir workers, and veterinarians are of higher risk of being exposed to the disease than others [11,12]. Furthermore, laboratory workers are in danger of contracting brucellosis through aerosols [13].

The main leading sign of brucellosis is abortion. Nonetheless, a vast physical examination is needed. Apart from that, several laboratory tests must take place to have a definitive diagnosis [14-16]. Since brucellosis can be transmitted to human in cattle’s milk and meat, it is considered as foodborne disease, so it is recommended to take certain precautions when handling animal products. Pasteurization is required to destroy the microorganisms found in milk and dairy products. Organs like meat must be handled cautiously as well [17-19].

According to the World Health Organization, Lebanon is experiencing an epidemiological transition, where infectious diseases have not disappeared yet. Brucellosis, for instance, is one of the zoonotic diseases that are considered as a major public health issue in eastern, southern, and northern areas. This country is home to Palestinian and Syrian refugees, especially in the region of Bekaa and South. As the number of people in shelters increases, safe drinking water and sanitation are becoming a major concern [20].

Lebanese Ministry of Public Health reported that Bekaa district has the highest human brucellosis incidence over the years with 300 cases per 1,000,000 individuals, followed by Mount Lebanon and South Lebanon. By the end of 2017, Southern Lebanon was considered an economic hub in the country being the second populated governorate (18.0% of the Lebanese population) [21]. A recent study showed that the highest rate of Brucella was found in the Bekaa region, which correlates with the epidemiological study in human cases in Lebanon [20]. On the other hand, there is no study done so far among farm animals in Southern Lebanon.

Thus, the aim of our study was to estimate the prevalence of *B. abortus* in South Lebanon using three different serological tests in the diagnosis of brucellosis in cattle.

## Materials and Methods

### Ethical approval

No animal was harmed during the sample collection. A licensed veterinarian was present with a comprehensive questionnaire that has been completed for each animal. This study was performed after requesting written consent from the Ministry of Agriculture and the Faculty of Agronomy and Veterinary Sciences in the Lebanese University for the collection and processing of samples.

### Study period, area and animals

**Figure-1:**
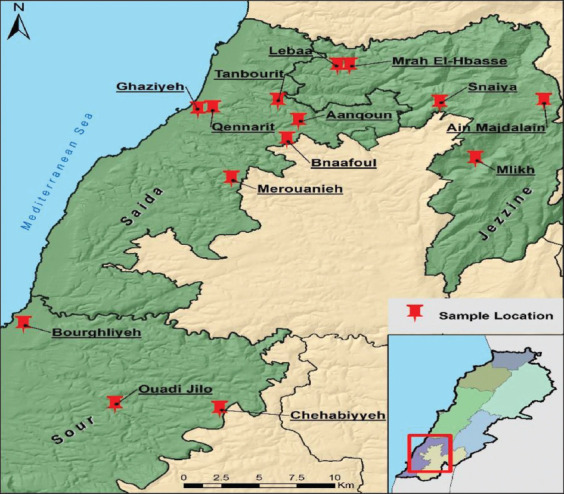
A map presenting the various regions chosen for the study.

This study was conducted from July 2019 to August 2019. Two hundred and three blood samples were collected from cattle of different ages. In addition, 121 bovine milk samples from cattle older than 2 years were collected from 15 farms in several rural regions of South Lebanon (Mlikh, Ain Majdalain, Sinaiya, Lebaa, Mrah El-Hbasses, Merouanieh, Ghaziyeh, Qennarit, Aanqoun, Bnaafoul, Tanbourit, Bourghliyeh, Chehabiyyeh, and Ouadi Jilo) (Figure-1). All selected farms were milk producers. A major factor to choose the farm was the presence of previous abortion cases. Non-vaccinated cattle were chosen for the study to prevent intervention with enzyme-linked immunosorbent assay (ELISA) results because an animal brucellosis vaccine contains the antigenic determinants that would be detected by ELISA. Few to no males were present in these farms due to the farmer’s choice of artificial insemination. The majority of the samples were taken from females. The samples were divided into districts (Jezzine, Saida, Tyre), gender (male and female), and age (below or above 2 years old).

### Sample size and power of the study

A prior statistical power analysis using GPower 3.1.9.2 software (Heinrich-Heine-Universität, Düsseldorf, Germany) revealed that the sample size n=203 was enough to attain a statistical power of at least 80% with alpha error of 5%, balanced on each side, and effect size of 0.2.

### Sample collection

Milk samples were taken from cows older than 2. The samples were taken manually from the udder and kept in sterile containers. The milk samples were preserved in the refrigerator at 4°C overnight before examination by milk ring test (MRT).

The blood samples were collected using 5 mL syringes, a 23 G needle from the subcutaneous abdominal vein in some cows and 18 G needle from jugular vein in calves, bulls, and rest of the cows. The blood samples were collected using the double-sided or one-sided needle, and the collected blood was vacated into vacutainers. After centrifugation, the serum was separated, extracted, and then stored in the freezer at −20°C before getting tested using different serological tests.

The following serological tests were used to detect the antibodies against *Brucella* antigens: Rose Bengal test (RBT), indirect ELISA (I-ELISA), and competitive ELISA (C-ELISA).

### MRT

MRT is an easy and fast screening method to detect brucellosis, yet multiple false results can occur. It detects immunoglobulin M (IgM) and IgA [22]. In this study, MRT produced by Central Veterinary Laboratory was used. As stated in the instructions, 1 mL of milk samples was taken using a micropipette and emptied into Eppendorf tubes. 0.03 mL of *B. abortus* antigen was added into the Eppendorf tubes with milk samples (Standardized *B. abortus*, and MRT Antigen - AHVLA - UK). Each tube of milk-antigen mixture was then incubated at 37°C for 1 h and examined afterward for the presence of a blue ring to indicate a positive reaction. In case of a negative result, the mixture remained homogeneous.

### RBT

RBT is a rapid screening agglutination test where stained *Brucella* suspension (acidic range) reacts with the serum [23]. The Pourquier^®^ Rose Bengal Antigen of *B. abortus* (Weybridge 99 strain) suspension manufactured by the IDEXX^®^ (Montpellier, France) was used. RBT has a higher affinity to the IgM [24]. Following the protocol, the serum was removed from the refrigerator and brought at room temperature. Thirty microliters of Rose Bengal solution was added to 50 μl of serum on a white glossy ceramic tile. The tile was then rocked at room temperature for 3 min. Any granulation formation is considered positive and then placed on the mechanical rotator for 3 min. Any granulation formation due to the reaction between the antigen and antibodies is considered positive. Otherwise, it is considered negative reaction.

### I-ELISA

The ELISA is another serological test that has been used worldwide to detect brucellosis. The I-ELISA is highly sensitive to *B. abortus* and *Brucella melitensis* strains. Its mechanism is based on the evaluation of the IgG antibodies. I-ELISA detects the secondary antibody that attaches to the serum antibody, which reacted with antigen-coated wells of the plate [25]. IDEXX^®^ Brucellosis Serum X2 ELISA Test kit was used for the study. The protocol provided by the manufacturer was followed. One hundred and ninety microliters of diluted wash solution was dispensed into each well of the plate to which 10 μL of undiluted serum was added, except for the first four wells that acted as control group. Positive control was added to wells numbered A1 and A2 while the negative control was added to wells B1 and B2. The mixtures were gently shaken. Afterward, the plate was covered and incubated overnight in the refrigerator at 4°C. The next day, all the wells were washed 3 times with 300 μL of the wash solution. Hundred microliters conjugate was distributed into each well of the plate. The plate was covered and incubated for 1 h at 37°C in a humid environment. The wells were washed again with the wash solution as previously done. Hundred microliters of 3,3’, 5,5”-tetramethylbenzidine substrate were distributed into each well and incubated for 15 min at 25°C. Hundred microliters of the stop solution were added to stop any reaction. The results were interpreted using the photometer at a wavelength of 450 nm.

### C-ELISA

The C-ELISA method requires antigen or anti­body-coated coated in the wells of the plate. A competition occurs between the tagged and untagged (serum tested) antigen or antibody to bind to the antigen or antibody present in the well [25]. The SVANOVIR *Brucella*-Ab C-ELISA kit was used for the positive samples for further confirmation. It is highly specific and discriminative between antibodies from vaccination and those from infection according to the manufacturer SVANOVIR^®^. All the materials were set at room temperature. Forty-five microliters of the “Sample Dilution Buffer” solution were dispersed in all the wells. Five microliters of the positive control, low positive, negative control, and the sample dilution buffer were added to the first wells in duplicates (A1, A2, B1, B2, C1, C2, D1, and D2, respectively). Five microliters of the sample serum were dispersed in the rest of the wells. Forty microliters of mAb-solution were added to the wells. Afterward, the plate was shaken in a plate shaker for 5 min, then covered and incubated at room temperature for ½ h. After incubation, the wells were washed and rinsed with the phosphate-buffered saline (PBS)-Tween buffer solution 4 times. Hundred microliters of the conjugate solution were added to wells and incubated at room temperature for ½ h. The wells were washed again with PBS-Tween buffer solution. Hundred microliters of the substrate solution were added and incubated for 10 min at room temperature. Fifty microliters of the stop solution were added to prevent any further reactions. The results were interpreted using the microplate photometer at a wavelength of 450 nm.

### Statistical analysis

Statistical analyses were conducted using the Statistical Package for the Social Science software (SPSS Statistics for Windows Version 22.0, IBM Corp., NY, USA). This software was used as well for data management and cleaning. Descriptive statistics were carried out and reported as frequencies and percentages for categorical variables. The Chi-square test was used to assess any significant difference between the categorical variables. The level of significance was set at p<0.05 for all statistical analyses. The ELISA used in this study allows for qualitative evaluation of the quality of humoral immunity (antibodies Ig). To validate the test, the means of optical density (OD) values of controls were calculated. For I-ELISA, the results were read as S/P ratio to determine the OD; hence, the equation was:


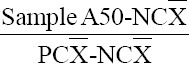



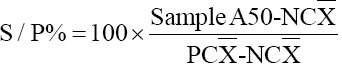
. If S/P ≥80%, result is negative; whereas if S/P ≤80%, result is positive. For C-ELISA, the percentage positivity value was calculated using the equation: 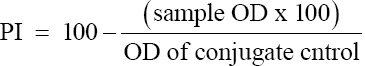. If PI <30%, result is negative and if PI ≥30%, result is negative. OD sample >1.0878 was considered negative and OD sample ≤1.0878 was considered positive in this study [26].

## Results

### Bovine brucellosis rate by MRT

**Figure-2:**
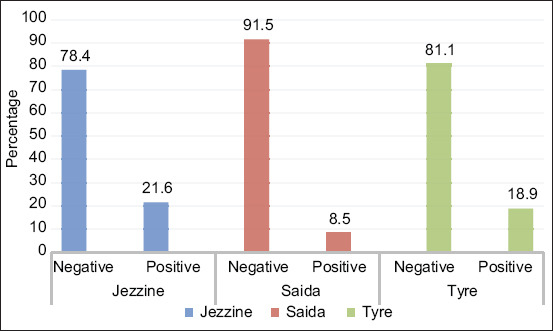
Percentage of positive and negative samples for each region using milk ring test.

Table-1 shows that the rate of brucellosis in bovine using MRT test varied across the three selected regions (Jezzine, Tyre, and Saida). Nineteen out of 121 (15.7%) (confidence interval [CI] 95 9.2-22.2%) milk samples came out as positive for brucellosis. All samples were females over 2 years old. According to the region, Jezzine exhibited the highest prevalence with 21.6% of the positive samples, followed by Tyre (18.9%) and Saida (8.5%) (Figure-2). The results showed a significant association between region and MRT test results (p<0.05). Bovine brucellosis in Jezzine was significantly higher than in Tyre and Saida.

**Table 1:** Results of MRT test in the three cities in Southern Lebanon.

MRT			

	Negative	Positive	p-value
Region (%)			0.012
Jezzine	29 (78.4)	8 (21.6)	
Saida	43 (91.5)	4 (8.5)	
Tyre	30 (81.1)	7 (18.9)	
Total	102 (84.3)	19 (15.7)	

MRT=Milk ring test

### Bovine brucellosis rate by RBT

**Figure-3:**
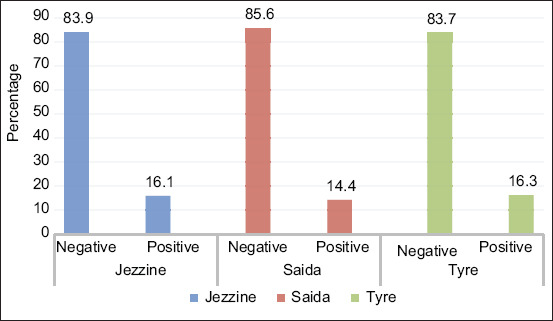
Percentage of positive and negative samples for each region using Rose Bengal test.

Table-2 summarizes the bovine brucellosis rate using RBT according to age, gender, and regions. Thirty-one blood samples out of 203 (15.3%) (CI 95 10.3-20.2%) were tested positive. All positive cases were females and over 2 years old. The samples taken from bovine aged <2 years were negative. According to the region, Figure-3 indicates that RBT showed a low variation of brucellosis among the three selected districts with more than 80% of samples being negative and a fluctuation between 14% and 16% of samples being positive. The results showed a significant association between region and test results (p<0.05). The prevalence of bovine brucellosis in Jezzine and Tyre was not significantly higher than in Saida.

**Table 2:** Results of RBTaccording to region, gender, and age.

RBT			

	Negative	Positive	p-value
Region (%)			0.005
Jezzine	47 (83.9)	9 (16.1)	
Saida	83 (85.6)	14 (14.4)	
Tyre	41 (83.7)	8 (16.3)	
Total	171 (84.7)	31 (15.3)	
Gender (%)			0.574
Female	166 (84.3)	31 (15.7)	
Male	5 (100)	0 (0.0)	
Total	171 (84.7)	31 (15.3)	
Age (%)			0.643
<2 years	4 (100)	0 (0.0)	
>2 years	167 (84.4)	31 (15.6)	
Total	171 (84.7)	31 (15.3)	

RBT=Rose Bengal test

### Bovine brucellosis rate by ELISA *B. abortus* indirect

**Figure-4:**
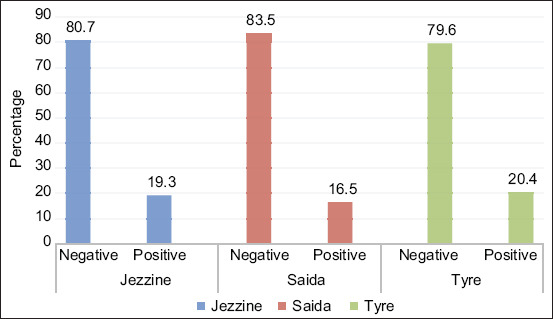
Percentage of positive and negative samples for each region using indirect enzyme-linked immunosorbent assay.

Table-3 displays the results acquired from testing with I-ELISA *B. abortus* according to age, gender, and regions. Thirty-seven out of 203 samples (18.3%) (CI 95 12.9-23.5%) were positive. According to gender, 18.2% of cows were positive and the rest were negative, while only one out of five bulls below 2 years old was tested positive. In other words, almost all positive samples were females. By looking at the positive samples in terms of district, Tyre and Jezzine showed the highest prevalence with 20.4% and 19.3%, respectively; whereas Saida followed with a prevalence of 16.5% (Figure-4). The results showed a significant association between region and I-ELISA test results (p<0.05).

**Table 3:** Results of I-ELISA test according to age, gender, and region.

I-ELISA			

	Negative	Positive	p-value
Region (%)			0.014
Jezzine	46 (80.7)	11 (19.3)	
Saida	81 (83.5)	16 (16.5)	
Tyre	39 (79.6)	10 (20.4)	
Total	166 (81.7)	37 (18.3)	
Gender (%)			0.934
Female	162 (81.8)	36 (18.2)	
Male	4 (80)	1 (20)	
Total	166 (81.7)	37 (18.3)	
Age (%)			0.597
<2 years	4 (80)	0 (20)	
>2 years	162 (81.4)	37 (18.6)	
Total	166 (81.7)	37 (18.3)	

I-ELISA=Indirect enzyme-linked immunosorbent assay

### Bovine brucellosis rate by indirect C-ELISA

C-ELISA was used on selected positive and negative samples for further confirmation. It is highly specific and highly discriminative between antibodies from vaccination and those from infection according to the manufacturer SVANOVIR^®^. Based on the previous results done by I-ELISA, only the positive samples selected from Jezzine and Tyre were tested for positive confirmation using C-ELISA, which corresponded to 75 serum samples. Fourteen of the 75 samples (18.7%) (CI 95 9.8-27.5%) were positive for brucellosis (Table-4). In Jezzine and Tyre, 19.6% and 15.8% of tested samples were positive for brucellosis, respectively. The results showed a significant association in terms of region (p<0.001). Among the three selected males, one was positive. The majority of positive cases were female above 2 years old.

**Table 4:** Results of C-ELISA test according to age, gender, and region.

C-ELISA			

	Negative	Positive	p-value
Region (%)			<0.001
Jezzine	45 (80.4)	11 (19.6)	
Tyre	16 (84.2)	3 (15.8)	
Total	61 (81.3)	14 (18.6)	
Gender n (%)			0.435
Female	59 (81.9)	13 (18.1)	
Male	2 (66.7)	1 (33.3)	
Total	61 (81.3)	14 (18.6)	
Age n (%)			0.235
<2 years	3 (100)	0 (0.0)	
>2 years	58 (80.5)	14 (19.4)	
Total	61 (81.3)	14 (18.6)	

C-ELISA=Competitive enzyme-linked immunosorbent assay

### Bovine brucellosis rate in South Lebanon according to 3 tests

**Figure-5:**
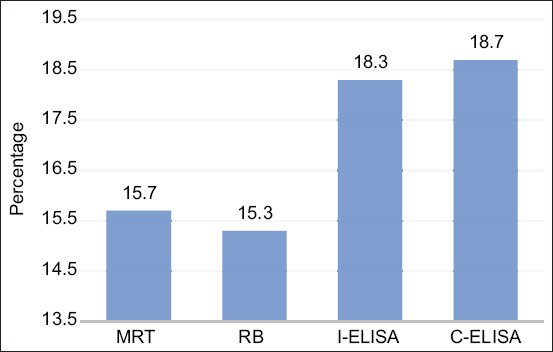
Percentage of total positive cases using three tests (Milk ring test, Rose Bengal, and enzyme-linked immunosorbent assay).

Figure-5 summarizes the positive rates of Brucellosis in South Lebanon according to the three tests. ELISA detected the highest percentage of positive samples for brucellosis, followed by MRT and RBT with slight variance between the positive samples. C-ELISA confirmed that the brucellosis rate in South Lebanon was around 18% (CI 95 9.8-27.5%) with a high prevalence found in Jezzine, followed by Tyre. Only one detected positive case was male and the rest were females older than 2 years.

## Discussion

Based on the previous studies, Brucellosis remains a serious endemic zoonotic disease in Lebanon and nearby countries. Our main research was to assess the prevalence of bovine brucellosis in different regions in South Lebanon. Three tests were performed to determine the rate of brucellosis: MRT, RBT, and ELISA.

The MRT is used widely by the farmers for fast results. Some studies confirmed the lower specificity and sensitivity compared to ELISA and RBT when it comes to confirming the presence of the *Brucella* infection. MRT screening can result in a higher number of false negatives due to presence of other diseases like mastitis, disorders like hormonal imbalances, different stages of lactation as in production of colostrum, late lactation stages or the presence of low IgM and IgA concentrations that are bound to milk fat globules [27,28].

The RBT has lower sensitivity than ELISA due to its inability to properly detect the IgG that exists in the chronic stage, hence, the need to follow-up with another serological test for a more accurate result. Factors that might affect the false negatives are the temperature that might alter the sensitivity and specificity of RBT, and the testing during the early stages of disease incubation or after abortion [28,29]. The possibility of false positives might occur in vaccinated cattle with the presence of IgM or in calves due to colostral antibodies [29].

As presented in Figure-5, ELISA detected 18.3% and 18.7% (I-ELISA and C-ELISA, respectively) positive cases, a higher percentage than both MRT (15.7%) and RBT (15.3%). In other studies, ELISA proved better and more accurate efficacy for the brucellosis detection, especially since it detects the late stages of the infection characterized by the presence of IgG mainly and IgA that slowly recedes. I-ELISA demonstrated high specificity and moderate sensitivity; whereas C-ELISA can demonstrate similar results, yet with the differentiation between vaccinated and non-vaccinated cattle [30-32].

In fact, our results go along with the previous studies that evaluated the three tests (MRT, RBT, and ELISA). Indeed, ELISA detected about 18% of positive cases; thus, a higher percentage of bovine brucellosis than those detected by MRT and RBT, which misclassified some results as false negative. Our results confirmed the presence of bovine brucellosis in South Lebanon and the three tests showed that Tyre and Jezzine had a higher significance in bovine brucellosis than Saida (Figures-2-4).

**Figure-6:**
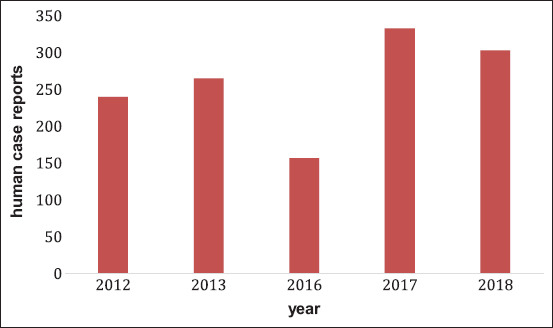
Human Brucellosis cases in Lebanon throughout the years.

All positive tests were of cattle above the age of 2 (Tables-3-5). It is reported that young animals might be more resistant to Brucellosis, while older animals tend to have lower immunity because of the lactating age. Heifers are less prone to brucellosis while sexually matured animals are more susceptible to it [33,34]. The majority of the positive samples were female. In most of the farms visited, artificial insemination was the farmer’s choice. Males were not common in farms where one to three can be present. Few farms on this study had their own bulls for insemination, one of them tested positive for brucellosis (Table-5). Through coitus, the male will infect the larger groups of females and continuously spreads the infection. Nonetheless, in some farms using artificial insemination, brucellosis was present that could be due to infected sperm or unhygienic insemination process. *Brucella* was found in most of the farms with a history of abortions. Yet some farmers tried to hide the occurrence or frequency of abortions in their farms in fear of production and economic loss that might be imposed on them. In majority of studies done, the seroprevalence of brucellosis was higher in females than in males as was seen by Adamu *et al*. [33], where females had higher seroprevalence than males by 13%. Females shed the disease more than males especially due to abortions and all discharges, and they are present for a longer period of time in the herd as was explained in a study by Kanouté *et al*. [34]. Approximately, all the cattle tested were of Holstein breed. Some studies proved that Holstein breed is more susceptible to brucellosis [35,36]. In Lebanon, human cases of Brucellosis were measured up to 300 cases in 2018 according to the Ministry of Public Health (Figure-6). Bekaa having the highest incidence among the 5 governorates of Lebanon followed by South, which reported five cases per 100,000 inhabitants in 2018 (Table-5). The persistence of human cases and the rise of cases in some governorates can be reflected on the incidences found by the animals. No study was done before in South Lebanon to compare animal incidence rate. Based on this study, animal and human incidence rate can be assumed in positive correlation as we can expect the continuous increase of one in relation to the increase of the other.

**Table 5:** Human case reports from the six governorates of Lebanon in the recent years.

Years	North	Beqaa	South-Lebanon	Mount-Lebanon	Beirut	Unknown
2011	31	52	18	24	1	8
2012	14	53	32	23	0	12
2013	21	78	36	20	3	31
2015	15	293	35	19	3	24
2016	25	286	42	22	4	23
2017	14	318	63	28	10	27
2018	14	89	64	37	21	17

An investigation stated that brucellosis has been persistent in Lebanon long before the Syrian crisis. Syria reported high incidences of brucellosis and increased especially during the Syrian war where there was a lack of veterinary control [37]. No reports were found about the vaccination programs for brucellosis in Syria. Consequently, many unvaccinated animals have been trespassing the Lebanese territory. This allows the spread of the disease among the livestock of Lebanese farmers. To this day, there still is deficient control and surveillance on the exchange and exporting of animals, that is, the animal movements between Lebanon and nearby countries, emphasizing on the lack of monitoring and screening process of possible disease carriers before the introduction of these animals into a herd [38].

All samples in this study were taken from small rural farms where extensive and semi-intensive livestock production mostly prevailed. According to Kalaajieh [3], the infection occurs more in the rural areas of Lebanon, that is, Bekaa and South regions where the tradition of consuming raw milk and dairy products with unhygienic and inappropriate handling is still evident. Indeed, the lowest human incidence in Lebanon is found in Beirut where little to no farms are found close by, and majority of people consume pasteurized and packaged dairy products [21]. In these smallholder dairy farms, low productivity, poor animal health, and hygiene can be attributed to the poor handling of husbandry practices. All animals tested were non-vaccinated due to insufficient governmental funds and the poor socioeconomic status of farmers. From discussions with farmers, we noticed lack of knowledge about brucellosis, and improper handling of infected cattle especially seen during the delivery where unhygienic means were practiced (handling of the fetus, abortus, and discharges). This allows other cows to come in contact with the waste, increases the spread of infection, and obstructs the control of brucellosis.

## Conclusion

Brucellosis is a zoonosis of bacterial origin, highly contagious that is transmitted through consumption of raw milk and dairy products and causes significant economic losses in Lebanon. Our results showed that bovine brucellosis is prevalent in Southern Lebanon. Ensuring animal health and medical prevention helps in containing the disease and reduces economic losses. As a start, sustainable surveillance and monitoring programs, in addition to a vaccination protocol, must be managed on farms, especially in those where raw milk is sold. For full control, several protocols must be implemented to educate consumer as well as farmers about the danger of brucellosis, about the fact that consuming raw milk is a route of infection, and about the importance of pasteurization and vaccination. Farmers and personnel should abide by Hazard Analysis and Critical Control Point practices to minimize the risk of infections that can be transmitted through direct contact, where these individuals are of high risk of spreading this disease [39,40]. They should also consider the necessary precautions in case of transportation or introduction of new animals to the farm. In Lebanon, the lack of consistent research and little feedback of occurring illness or symptoms create a significant gap in helping to control the spread of the disease. Further studies must be conducted in regards to the differentiation between *B. abortus* and *B. melitensis* in cattle to have a more defined insight on bovine brucellosis in our country. In addition, further studies regarding the prevalence, risk factors and antibiotic resistance profiles of different *Brucella* species must be done among Lebanese citizens.

## Authors’ Contributions

HH and RA designed the study and wrote the manuscript. GG collected the samples and helped in the laboratory work. AS carried out the statistical analysis and helped in writing the manuscript. NN and JE helped in the laboratory work. RA did the laboratory analysis. GG and AS contributed equally to the work. All authors read and approved the final manuscript.
